# What Works? Improving Cardiometabolic Health in Our Patients – A Full Audit Cycle

**DOI:** 10.1192/bjo.2025.10382

**Published:** 2025-06-20

**Authors:** Claire Jones, Jade Evans, Emily Sergeant, Maura Killoughery

**Affiliations:** 1 South West London and St George’s Mental Health NHS Trust, London, United Kingdom; 2 Kingston and Richmond NHS Foundation Trust, London, United Kingdom

## Abstract

**Aims:** People under 75 years in contact with secondary mental health services have a significantly higher mortality and morbidity. Psychiatric medications increase the risk of cardiometabolic syndrome, psychiatric patients present less for attention of their physical health needs and are more likely to be overweight/smoke.

The aim of the Cardiometabolic Assessment (CMA) Clinic in our community mental health team is to reduce health inequality – identifying physical health problems, escalate any issues for medical treatment/review (usually to GP) and ensure patients receive treatment or get signposted for health promotion services.

We reviewed our clinic, identifying if it was fulfilling this purpose and looked at did not attend (DNA) rates.

Audit standards were that all patients invited to the CMA clinic should attend and that the CMA clinic should appropriately escalate all patients with concerning physical observations or requiring signposting to additional services.

**Methods:** Retrospective audit review of 247 appointments, January–December 2023.

We assessed: DNA rates; Physical health issues identified; Appropriate escalation and/or signposting e.g. for stop smoking services or obesity.

Change was implemented:

Educating staff with group and 1:1 teaching (colleagues from primary care were invited).

Written information leaflet sent with each appointment describing importance/purpose of CMA clinic.

Developed a simple proforma based on the modified early warning scoring system for CMA staff to send to the GP.

Staff granted the ability to directly contact GP practices.

Re-audited 36 appointments in the clinic from May–July 2024.

**Results:** Initial audit cycle: DNA rate of 52%. 53% of physical health issues managed correctly.

Re-audit: DNA rate 42%. 90% of physical health issues managed correctly.

Re-audit showed improvements in both standards.

**Conclusion:** A small intervention has enabled a direct improvement in patient care.

The DNA rate remains high – potentially a more focused approach to patients attendance may be beneficial. After ongoing multidisciplinary discussions we recommend that CMA clinic staff should consider domiciliary visits for patients at high risk of cardiometabolic conditions who repeatedly fail to attend CMA.

Clinicians to use all opportunities for CMA monitoring and make every contact count for physical health including occurring during care-coordinator reviews, in depot or other clinics. This will reduce the number of times a patient needs to visit the department hopefully improving satisfaction and increasing attendance.

Continued joint working with colleagues in primary care so workload is not duplicated and all opportunities for patient contact is maximised with the aim of reducing morbidity and mortality.

